# Supplementary material for: SigProfilerMatrixGenerator: a tool for visualizing and exploring patterns of small mutational events
Source: BMC Genomics. 2019 Aug 30;20:685. doi: 10.1186/s12864-019-6041-2 (PMC6717374; doi:10.1186/s12864-019-6041-2)
Supplement: Supplementary file 3 — Figure S1. Performance for matrix generation across six commonly used tools. (DOCX 2606 kb) [file 12864_2019_6041_MOESM3_ESM.docx]

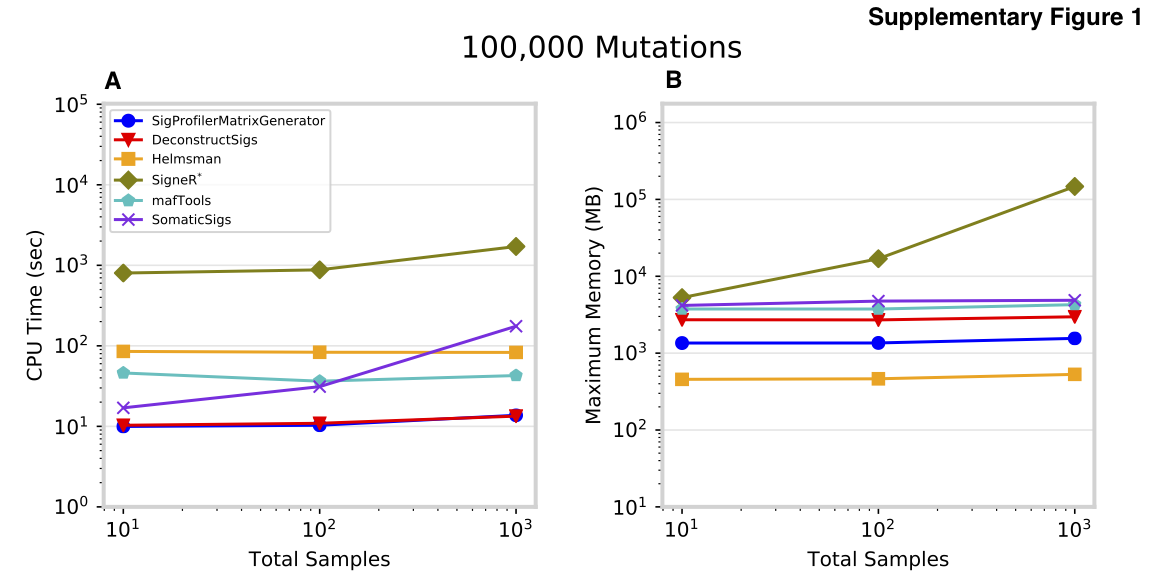


**Figure S1: Performance for matrix generation across six commonly used tools.** Each tool was evaluated separately using 10, 100, and 1,000 VCF files, each corresponding to an individual cancer genome, containing a total of 100,000 somatic mutations ***A)*** CPU runtime recorded in seconds (log-scale) and ***B)*** maximum memory usage in megabytes (log-scale). Performance metrics exclude visualization.
